# Induction chemotherapy in patients with resectable head and neck squamous cell carcinoma: a meta-analysis

**DOI:** 10.1186/1477-7819-11-67

**Published:** 2013-03-14

**Authors:** Jie Ma, Ying Liu, Xi Yang, Chen-ping Zhang, Zhi-yuan Zhang, Lai-ping Zhong

**Affiliations:** 1 Department of Oral Maxillofacial-Head Neck Oncology, Ninth People’s Hospital, Shanghai Jiao Tong University School of Medicine, Shanghai Key Laboratory of Stomatology, No.639 Zhizaoju Rd, Shanghai, 200011, China

**Keywords:** Induction chemotherapy, Resectable head and neck squamous cell carcinoma, Randomized controlled trial, Meta-analysis

## Abstract

**Background:**

Induction chemotherapy has been investigated as a possible strategy to shrink or downstage locally advanced head and neck cancers, providing opportunity to remove the lesions completely after induction chemotherapy, especially in the patients with resectable advanced disease. The aim of this study was to investigate the definitive effect of induction chemotherapy in patients with resectable head and neck squamous cell carcinoma.

**Methods:**

A meta-analysis of randomized trials (1965–2011) was performed on the impact of induction chemotherapy on survival, disease control, and toxicity in this population of patients. Kaplan-Meier curves were read by Engauge-Digitizer. Data combining was performed using RevMan.

**Results:**

Fourteen trials (2099 patients) were involved in this analysis. There was no significant difference on overall survival, disease free survival, or locoregional recurrence between the patients treated with and without induction chemotherapy (*P* >0.05). However, the patients treated with induction chemotherapy had a lower rate of distant metastasis by 8% (95% confidence interval 1%–16%, *P* = 0.02) than those treated without induction chemotherapy. In patients with laryngeal cancer, comparing to radical surgery, the larynx could be preserved in responders to induction chemotherapy without survival decease (*P* >0.05). Induction chemotherapy-associated death was 0%–5%.

**Conclusions:**

Based on the results above, there is a significant benefit of induction chemotherapy on decreasing distant metastasis in patients with resectable head and neck squamous cell carcinoma. In patients with laryngeal cancer, induction chemotherapy provides larynx preservation in responders to induction chemotherapy.

## Background

Head and neck squamous cell carcinoma (HNSCC) ranks sixth among the most common cancers worldwide with an incidence of over 500,000 new cases each year [1]. Patients at an early stage clinical (stages I and II), are typically treated with single modality therapy, usually surgery or radiation therapy, with excellent disease control and long-term survival. For patients with more advanced disease, at clinical stages III and IV, comprehensive, sequential treatment regimens consisting of surgery, and/or radiotherapy, with or without chemotherapy are mostly required [2]. However, the prognosis has not been significantly improved; the 5-year survival rate remains about 50% to 60%, and is even lower in the patients at late clinical stages [3].

Induction chemotherapy has been investigated as a possible strategy to shrink or downstage locally advanced head and neck cancers, increase organ preservation rates, and/or reduce the risk of locoregional and/or distant recurrence, ultimately improving treatment outcomes. However, it should be noted that after induction chemotherapy locoregional treatment should be performed according to the original tumor borders, which should be marked with a tattoo or ink before treatment initiation; for example, after preoperative induction chemotherapy, in order to remove the tumor completely, surgical resection should be performed according to the original tumor borders, regardless of the response to induction chemotherapy. Drug delivery is postulated to be better in untreated well-vascularized tumors before surgery and/or radiotherapy than that after surgery and/or radiotherapy. However, there is still debate about the clinical value of induction chemotherapy, especially for resectable HNSCC. Both positive and negative results from randomized controlled trials (RCTs) have been reported, and previous systematic reviews of these results have not merited a clear-cut benefit of induction chemotherapy on overall survival [4-7]. For locally advanced and resectable HNSCC, there is still no systemic analysis of the outcomes of induction chemotherapy followed by locoregional treatment compared to locoregional treatment alone, thus, we performed a meta-analysis of survival rate, locoregional control, distant metastasis, and toxicity.

## Methods

### Eligibility criteria

RCTs were eligible if they were studies of previously untreated patients with resectable non-metastatic HNSCC, comparing induction chemotherapy followed by locoregional treatment (comprising surgery, or radiotherapy or chemoradiotherapy, or surgery plus radiotherapy or chemoradiotherapy) versus locoregional treatment. The RCTs for larynx preservation were also eligible if they had compared radical surgery and radiotherapy versus induction chemotherapy, followed by radiotherapy or chemo-radiotherapy in responders, or radical surgery and radiotherapy or chemoradiotherapy in non-responders. The RCTs were limited to those officially published in English, and based on patients recruited between 1 January 1965 and 31 December 2011. Tumor sites included the oral cavity, oropharynx, hypopharynx, and larynx with the exception of the nasopharynx.

### Search strategy

Literature searching was conducted using the database of MEDLINE from 1965 to 2011 and EMBASE from 1980 to 2011. Reference lists and conference proceedings were also searched to identify possible additional RCTs. The following search codes were used: induction chemotherapy.tw, induc$ chemotherapy.tw, neoadjuvant chemotherapy.tw, preoperative chemotherapy.tw, sequential chemotherapy.tw, adjuvant chemotherapy.tw, primary chemotherapy.tw, initial chemotherapy.tw, resectable.tw, operable.tw, head and neck.tw, oral.tw, pharyngeal.tw, oropharyngeal.tw, hypopharyngeal.tw, maxillofacial.tw, laryngeal.tw, paranasal sinus.tw, randomized controlled trials/, randomised-controlled-trial.pt, controlled-clinical-trial.pt, random allocation/, exp clinical trials/, clinical-trial.pt, random$.ti,ab, comparative study/, follow-up studies/, prospective studies/.

### Data collection and analysis

Suitability of studies for inclusion was independently assessed by two authors and any disagreement or lack of clarity was resolved through discussion. We developed a data extraction sheet based on the Cochrane Consumers and Communication Review Group data extraction template. The data, including patient number, age, sex, tumor site, tumor, node, metastasis (TNM) stage, were also extracted and checked by the two authors and disagreement was resolved through discussion. If agreement could not be reached between the two authors, a third author would participate in the discussion and so on, until reaching final agreement. The primary endpoint was overall survival. The secondary endpoints were disease-free survival, locoregional recurrence, and distant metastasis.

The time-to-event data from individual trials were summarized by the log hazard ratio (HR) and its variance. If the trials did not report this information directly, appropriate data, such as the *P*-value from the log-rank test were extracted to estimate the log HR and its variance [8], and the time-to-event data were extracted from the survival curves. Kaplan-Meier curves were read by the Engauge Digitizer version 4.1 (free software downloaded from http://sourceforge.net). Data combining was per-formed by RevMan version 5.1 (free software downloaded from http://www.cochrane.org). The log HR and its variance were pooled using an inverse variance weighted average, and the results were presented as an HR and 95% CI.

DerSimonian-Laird random effect analysis was used to estimate the survival difference [9]. This method generates a combined survival difference and a 95% CI with a heterogeneity test at each endpoint. Survival rate was derived from published survival curves if it was not provided explicitly in the text or tables. Subjects censored prior to each endpoint were subtracted from the denominators (number of patients during follow-up), giving a conservative CI for the summary statistic. Censored cases were counted by placing tick marks on survival curves when provided [10].

Heterogeneity was assessed by inspection of the forest plot, the Cochran chi-squared (*χ*^2^) test, and the *I*^2^ statistic percentage. A fixed effect approach was adopted unless there was significant evidence of unexplained heterogeneity, in which case a random effects approach was used.

## Results

**Figure 1:**
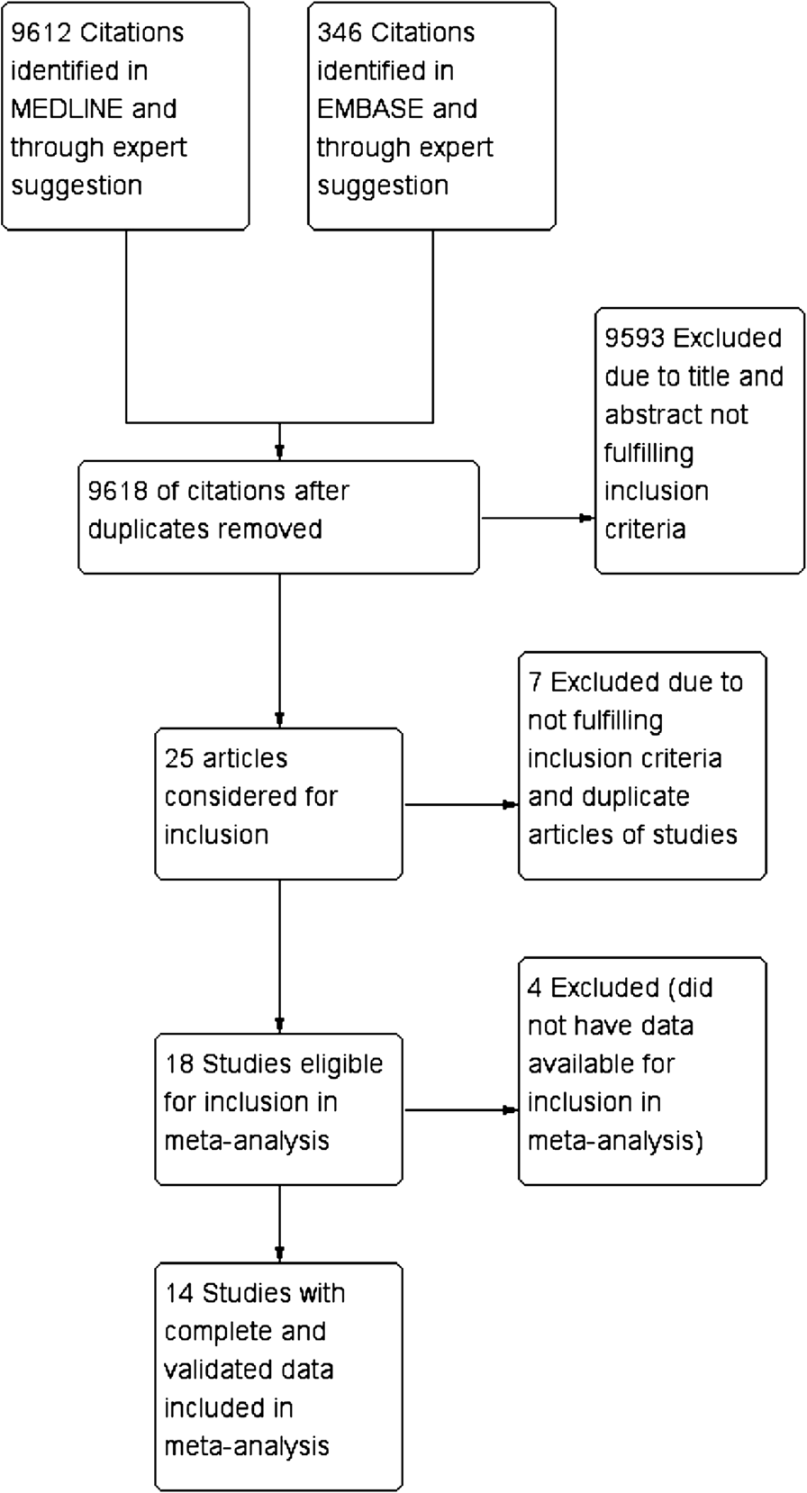
Study flow diagram.

A total of 9,612 citations were identified from the database of MEDLINE and EMBASE, and there were 18 RCTs that fulfilled the inclusion criteria. After review by all of the authors, 14 RCTs (2,107 patients) [11-27] were found to be eligible with complete and validated data for meta-analysis (Figure 1).

Among the 14 RCTs, comparison between the patients receiving induction chemotherapy followed by locoregional treatment (surgery and/or radiotherapy or chemo-radiotherapy), and locoregional treatment alone (surgery and/or radiotherapy or chemoradiotherapy) was reported in 11 RCTs (1,505 patients) [11-23]. Comparison between induction chemotherapy followed by surgery in non-responders or radiotherapy/chemoradiotherapy in responders, and radical surgery and radiotherapy was reported in three RCTs, focusing on larynx preservation (602 patients) [24-27]. Although there were a few variations in these trials, such as period of study and duration of follow-up, the influence of study heterogeneity on the relative risk of disease-specific death was not significant (*I*^2^ = 0%, *P* = 0.54). According to the toxicity of induction chemotherapy as reported in the trials, the most common toxicity effect was vomiting (8.5% to 24.5%), followed by leukopenia (5.1% to 7.6%), mucositis (0.2% to 8.2%), and thrombocytopenia (1.7% to 7.7%); the induction chemotherapy-associated death rate was reported to be 0% to 5%.

**Figure 2:**
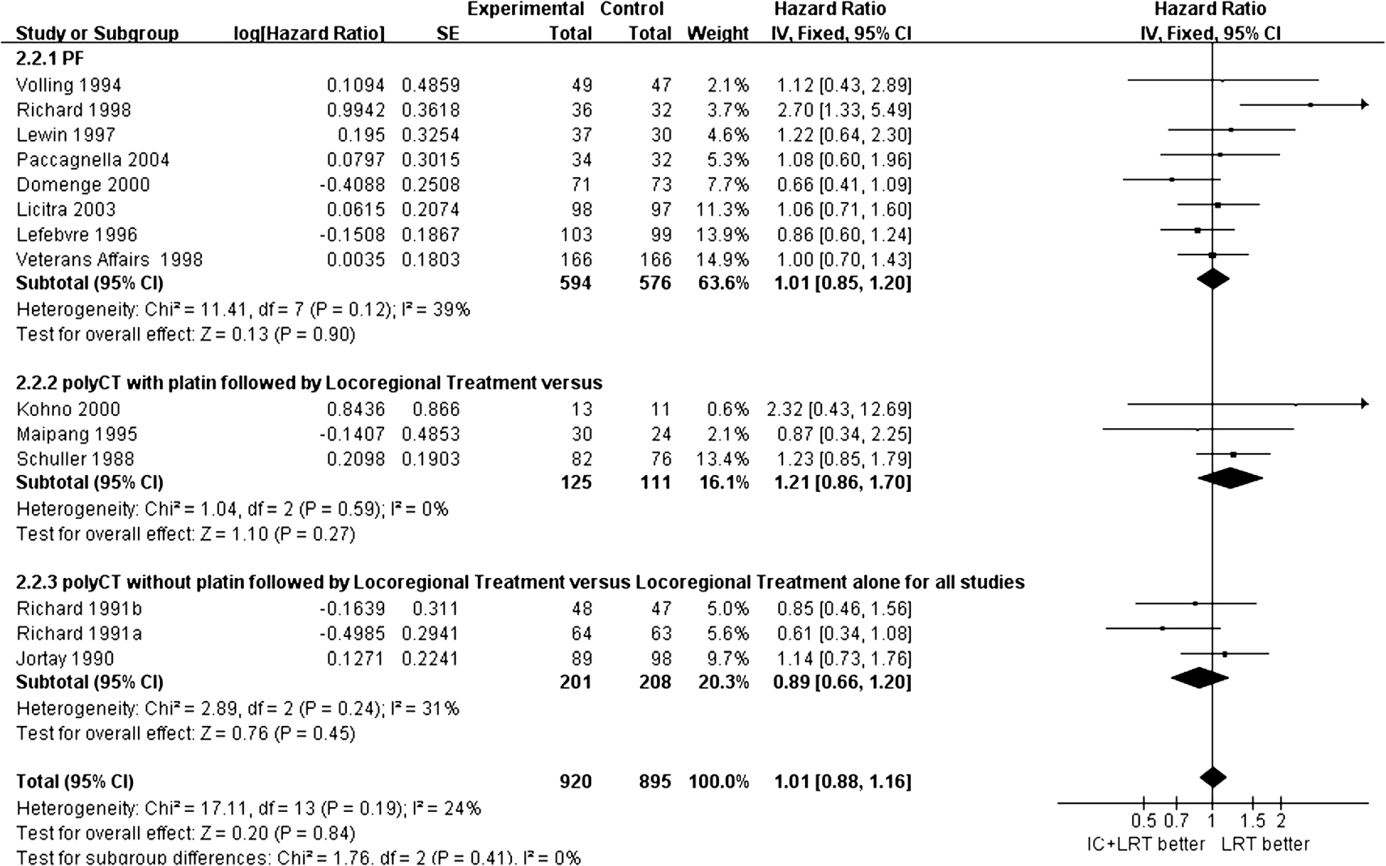
**Forest plot of hazard ratios for overall survival and 95% CI in fourteen randomized controlled trials in patients with resectable head and neck squamous cell carcinoma treated with induction chemotherapy followed by locoregional treatment, or locoregional treatment alone.** Richard 1991a^23^ is for cancer of the floor of the mouth, Richard 1991b^23^ is for cancer of the posterior oral cavity and oropharynx. Volling 1994^13^, Richard 1998^27^, Lewin 1997^14^, Paccagnella 2004^11^, Domenge 2000^15^, Licitra 2003^16^, Lefebvre 1996^24^, Veterans Affairs 1998^27^, Kohno 2000^21^, Maipang 1995^19^, Schuller 1988^20^, Jortay 1990^22^.

**Figure 3:**
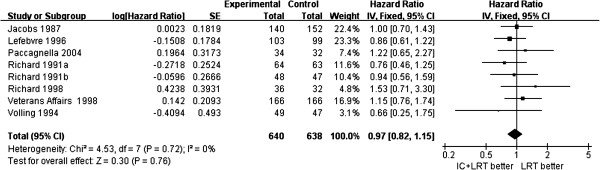
**Forest plot of hazard ratios for disease-free survival and 95% CI in patients with resectable head and neck squamous cell carcinoma treated with induction chemotherapy followed by locoregional treatment, or locoregional treatment alone.** Richard 1991a^23^ is for cancer of the floor of the mouth, Richard 1991b^23^ is for cancer of the posterior oral cavity and oropharynx. Jacobs 1987^17^, Lefebvre 1996^24^, Paccagnella 2004^11^, Richard 1998^25^, Veterans Affairs 1998^27^, Volling 1994^13^.

There was no significant difference in overall survival between patients treated with and without induction chemotherapy (HR = 1.01, 95% CI 0.88, 1.16, *P* = 0.84), neither was there a significant difference according to the protocol of induction chemotherapy, such as cisplatin and 5-fluorouracil (PF), other platin-containing combinations, or multiple agents without platin (Figure 2). For disease-free survival, there was no significant difference between the patients treated with or without induction chemotherapy (HR = 0.97, 95% CI 0.82, 1.15, *P* = 0.76) (Figure 3).

In the three RCTs of laryngeal or hypopharyngeal cancer (602 patients) focusing on larynx preservation [24-27], compared to radical surgery followed by radiotherapy, the larynx could be preserved after induction chemotherapy in responders without decrease of overall survival (HR = 1.21, 95% CI 0.72, 2.03, *P* = 0.47) or disease-free survival (HR = 1.02, 95% CI 0.79, 1.31, *P* = 0.87).

**Figure 4:**
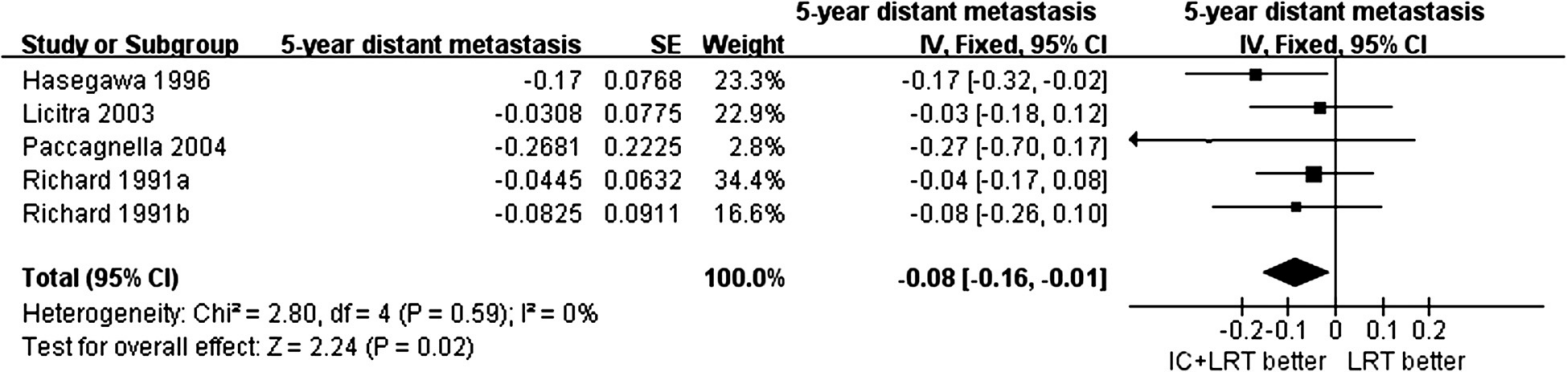
**Forest plot of hazard ratios of distant metastasis-free survival and 95% CI in patients with resectable head and neck squamous cell carcinoma treated with induction chemotherapy followed by locoregional treatment, or locoregional treatment alone.** Richard 1991a^23^ is for cancer of the floor of the mouth, Richard 1991b^23^ is for cancer of the posterior oral cavity and oropharynx. Hasegawa 1996^18^, Licitra 2003^16^, Paccagnella 2004^11^.

There was no significant difference in long-term (5-year) locoregional recurrence rate between patients treated with or without induction chemotherapy (432 patients, ratio difference = 2%, 95% CI −12%, 16%, *P* = 0.76). However, among patients who developed distant metastases (700 patients), those treated with induction chemotherapy had a significantly lower long-term (5-year) rate of distant metastases (8% difference, 95% CI 1%, 16%, *P* = 0.02), compared to those treated without induction chemotherapy (Figure 4).

## Discussion

In this study, for patients with locally advanced and resectable HNSCC, induction chemotherapy benefited these patients in terms of an 8% lower rate for the occurrence of distant metastases; however, induction chemotherapy did not improve overall survival, disease-free survival or locoregional control. The toxicity of induction chemotherapy was acceptable for further surgery, or radiotherapy or chemoradiotherapy.

Previous meta-analyses of both resectable and unre-sectable HNSCC [4-7] and this meta-analysis of resectable HNSCC confirm the effective decrease in the development of distant metastases in the patients without metastases at baseline (M0) treated with induction chemotherapy and locoregional treatment, compared to locoregional treatment alone. This is reasonable due to the effect of induction chemotherapy on peripheral, potentially metastatic tumor cells.

In this study, with respect to overall survival, we found no significant benefit of induction chemotherapy in the patients with resectable HNSCC, even using the PF protocol, which has been reported to be beneficial to overall survival in other meta-analyses [4,6,7]. According to the site of primary lesions, most of patients receiving PF induction chemotherapy in the present study were oral cancer patients; while the previous studies of induction chemotherapy in HNSCC patients included not only oral cancer, but also patients with oropharyngeal and hypopharyngeal cancer. As we know, for oral cancer patients with resectable lesions, radical surgery is considered as the standard of care, followed by post-operative radiotherapy or chemoradiotherapy, depending on the presence of intermediate/high risk features in the surgical specimen; and for patients with resectable locally advanced oropharyngeal and hypopharyngeal cancer, surgery, or radiotherapy or chemoradiotherapy, followed by radiotherapy or chemoradiotherapy is considered the standard of care. The efficiency of adding PF agents to standard care may differ between patients with oral cancer, and those with oropharyngeal or hypopharyngeal cancer. So, the effect of PF induction chemotherapy may differ in the patients with different primary tumor sites, and therefore, the primary tumor site might be considered before adding PF induction chemotherapy. It appears that induction chemotherapy could be more effective in oropharyngeal and hypopharyngeal cancer than in oral cancer. This could be due to various factors, such as high-risk human papilloma virus (HPV) infection, producing virus oncoproteins of E6 and E7, which are necessary for viral replication through their proliferation-stimulating activity, and play a key role in malignant transformation and maintenance; they are also sensitive to cytotoxic chemotherapy and DNA damage-induced apoptosis. Also, as we know, the incidence of HPV in patients with oropharyngeal cancer is higher than in patients with oral cancer. Based on the results of the present study, some factors might be considered to improve the prognosis in future clinical trials, such as insistence on radical surgery, even in patients with clinical response, in order to reduce locoregional failure and to improve survival, or optimization of the induction chemotherapy protocol by adding new or targeted drugs.

Recently, in two randomized phase III trials [3,28,29], a new induction chemotherapy protocol of a combination of docetaxel, cisplatin and 5-fluorouracil (TPF) followed by radiotherapy or chemoradiotherapy has been shown to improve survival compared to PF, and it is suggested as the preferred chemotherapy regimen when induction treatment is used for management of HNSCC patients. However, there is still little evidence from large clinical trials that the use of induction TPF prior to locoregional treatment improves survival when compared to locoregional treatment alone. Furthermore, it is unknown whether induction TPF improves outcomes when given prior to surgery in patients with locally advanced and resectable HNSCC. Several clinical trials of epidermal growth factor receptor inhibitors suggest the targeted drugs could improve locoregional control and survival in HNSCC patients with primary, recurrent or distant metastatic lesions; cetuximab, for example, is now firmly established as an active component of treatment for advanced HNSCC, alone and in combination with other modalities, including radiotherapy, platinum-based chemotherapy and induction therapy. Cetuximab has been approved by the Food and Drug Administration (FDA) USA, for HNSCC treatment in combination with radiotherapy for locally advanced, potentially curable disease, and as a single agent for incurable recurrent or metastatic disease [30-33]. Additional clinical trials are warranted to determine the benefit of adding an epidermal growth factor receptor (EGFR)-targeted agent in the setting of locally advanced and resectable HNSCC.

As we know, the different response to induction chemotherapy could lead to different survival, with good response always leading to good survival, bad response leading to poor survival [16]. Some predictive biomarkers reflecting the response to induction chemotherapy could be helpful for the next treatment choice, or in deciding whether induction chemotherapy should be performed, especially for resectable lesions. If the individual biomarkers predict bad response to induction chemotherapy, it should not be performed in those patients; otherwise, induction chemotherapy could benefit both patient response and survival. The biomarkers include DNA sequence mutations, epigenetic changes, and levels of messenger RNA or protein expression. For example, in a prospective study [34], *p53* gene mutations are strongly associated with a poor risk of both objective and major response to PF-based induction chemotherapy, suggesting that patients with HNSCC should first be screened for *p53* mutations, before choosing the most appropriate treatment protocol based on the mutations.

For organ preservation, it has been well-recognized that in patients who respond to it, induction chemotherapy, followed by radiotherapy or chemoradiotherapy, instead of radical surgery, could benefit patients with laryngeal cancer by preserving the larynx, without a negative impact on overall survival and disease-free survival. For other organs, although the response rate to induction chemotherapy is relative high (50% to 80%) in the resectable lesions, which provides a better chance to eradicate the locoregional lesions by radical surgery, there is no conclusive evidence that induction chemotherapy confers the benefit of organ preservation. A report by Licitra *et al*. [16] revealed that induction chemotherapy can reduce the number of patients requiring mandibulectomy and/or radiation therapy. However, there are no further reports on the differences in survival or locoregional recurrence between patients who do or do not undergo mandibulectomy and/or radiotherapy. In our opinion, in order to remove the tumor completely, emphasis should placed on surgical resection being performed according to the original tumor borders, which are marked with tattoo or ink before treatment initiation, regardless of the response to induction chemotherapy. In this study, there was no evidence of significant differences in relation to benefit for locoregional control between patients receiving or not receiving induction chemotherapy. Further trials are needed to resolve whether induction chemotherapy can lead to organ perseveration of non-laryngeal sites.

## Conclusions

In conclusion, there is a significant benefit of induction chemotherapy in reducing distant metastases in patients with locally advanced and resectable HNSCC; however, there is no strong evidence of benefit in survival or locoregional control. In contrast, induction chemotherapy can be quite effective for preservation of the larynx. Further research on non-laryngeal organ preservation are encouraged using optimized induction chemotherapy protocols, and also evaluating molecular biomarkers that could help to identify those patients most likely, or unlikely to benefit from the addition of induction chemotherapy to their treatment regimen.

## Abbreviations

EGFR: epidermal growth factor receptor; FDA: Food and Drug Administration; HNSCC: head and neck squamous cell carcinoma; HPV: human papilloma virus; RCT: randomized controlled trial; HR: hazard ratio; PF: cisplatin and 5-fluorouracil; TNM: tumor node, metastasis; TPF: docetaxel cisplatin and 5-fluorouracil.

## Competing interests

The authors declare that they have no competing interests.

## Authors’ information

Co-corresponding author: Zhi-yuan Zhang.

## Authors’ contributions

LZ and ZZ participated in the study design and coordination. CZ participated in the study coordination. JM, YL and XY participated in the literature search, data extraction and statistical analysis. All authors edited the manuscript and approved the final manuscript.
